# Blocking MyD88 Signaling Pathway Protects Against Myocardial Ischemia Reperfusion by Inhibiting NOX/ROS Pathway and Enhancing eNOS Activity

**DOI:** 10.3390/ijms27188299

**Published:** 2026-09-17

**Authors:** Bo Wang, Xia Huang, Lin Xie

**Affiliations:** 1 Department of Gastroenterology, Tongji Hospital, Tongji Medical College, Huazhong University of Science and Technology, Wuhan 430074, China; wangbo@tjh.tjmu.edu.cn; 2 Institute of Organ Transplantation, Tongji Hospital, Tongji Medical College, Huazhong University of Science and Technology; Key Laboratory of Organ Transplantation, Ministry of Education; NHC Key Laboratory of Organ Transplantation; Key Laboratory of Organ Transplantation, Chinese Academy of Medical Sciences; Hubei Provincial Clinical Research Center for Organ Transplantation, Wuhan 430030, China; xiahuang@thj.tjmu.edu.cn

**Keywords:** myocardial, ischemia–reperfusion injury, MyD88 signaling, ROS, NOX, NADPH, eNOS, Innate immunity, Redox homeostasis

## Abstract

Myocardial ischemia–reperfusion injury (MIRI) remains a major complication in acute coronary syndrome and cardiac surgery, with oxidative stress and metabolic dysregulation serving as central pathogenic drivers. This study aimed to clarify whether the novel MyD88 inhibitor TJ-M2010-5 confers cardioprotection against MIRI by remodeling immune–metabolic homeostasis, particularly through regulating NADPH oxidase (NOX)-derived reactive oxygen species (ROS) overproduction and endothelial nitric oxide synthase (eNOS) functional activity. In this study, male C57BL/6 mice were subjected to 30 min myocardial ischemia followed by 24 h reperfusion, with or without TJ-M2010-5 pretreatment (50 mg/kg, i.p.), and mouse cardiac vascular endothelial cells (H5V) were exposed to hypoxia–reoxygenation (H/R) with or without TJ-M2010-5 intervention. We systematically evaluated myocardial infarct size, ROS production, cell apoptosis, NF-κB activation, NOX expression and activity, NADPH redox homeostasis, NO production, and eNOS phosphorylation levels. The results showed that TJ-M2010-5 significantly reduced myocardial infarct size and inhibited IKKβ/NF-κB activation in I/R-treated mice, and functionally disrupted MyD88 homodimerization in ischemic myocardium. In H/R-challenged H5V cells, TJ-M2010-5 decreased ROS generation and cell apoptosis, suppressed NOX2/NOX4 expression and activity, restored NADPH levels and NADP^+^/NADPH redox balance, preserved tissue NO content, and maintained eNOS Ser1177 phosphorylation. In conclusion, pharmacologic blockade of MyD88 by TJ-M2010-5 alleviates NOX-dependent oxidative stress, stabilizes NADPH redox homeostasis, preserves NO bioavailability, and maintains eNOS functional activity, thereby exerting potent cardioprotective effects against MIRI via regulating redox homeostasis. The TLR/MyD88–NOX/ROS–eNOS signaling axis represents a novel and promising redox-regulatory therapeutic target for the clinical prevention and treatment of MIRI.

## 1. Introduction

Myocardial ischemia reperfusion injury (MIRI) is a common and serious complication following cardiac surgery or treatment for myocardial infarction, significantly affecting patient prognosis. Reactive oxygen species (ROS) play a key role in MIRI [1]. Under normal physiological conditions, ROS serve as important signaling molecules, with low levels participating in various essential processes such as cell proliferation, differentiation, apoptosis and intercellular signal transduction [2]. However, when endothelial cells and cardiomyocytes are subjected to oxidative stress or other stimuli, ROS levels in cardiac tissue rise substantially, disrupting the intracellular redox balance. Massive ROS accumulation triggers genomic instability, DNA damage, and inflammatory responses, ultimately resulting in mitochondrial dysfunction and cardiovascular disease [3].

Emerging evidence indicates that disrupted cellular metabolic homeostasis, especially nicotinamide adenine dinucleotide phosphate oxidase (NADPH oxidase, NOX)-dependent redox metabolic dysfunction, is a critical but insufficiently explored pathogenic mechanism underlying MIRI. NADPH serves as a central metabolic node, functioning both as an electron donor for NOX-mediated ROS generation and as an essential cofactor for eNOS-dependent nitric oxide production. Under pathological conditions, the redistribution of NADPH flux from physiological antioxidant pathways toward pathological ROS production drives metabolic collapse and exacerbates tissue injury.

NOX is a key enzyme responsible for intracellular ROS production. NOX is a family of transmembrane oxidoreductases whose primary function is to transfer electrons from NADPH on one side of the membrane to oxygen molecules on the other, thereby generating ROS—predominantly superoxide anion and hydrogen peroxide. The NOX family includes multiple subtypes (e.g., NOX1-NOX5, DUOX1 and DUOX2) with distinct tissue-specific expression patterns and biological functions [4,5]. In cardiac tissue, NOX2 and NOX4 are the predominantly expressed functional isoforms closely associated with myocardial pathological remodeling [6]. NOX2 activation is involved in angiotensin II-mediated myocardial hypertrophy and fibrosis in mice [7,8]. Nox2^−/−^ mice displayed reduced cardiomyocyte hypertrophy, apoptosis, and interstitial fibrosis following myocardial infarction [9]. Meanwhile, NOX4 promotes cardiomyocyte apoptosis via H_2_O_2_ production and mediates H_2_O_2_-dependent fibroblast proliferation, an important mechanism in the development of heart failure [10]. Sustained NOX-dependent ROS overproduction induces persistent oxidative stress and is closely associated with the pathogenesis and progression of cardiovascular diseases, including MIRI [11,12]. Thus, strategies that target oxidative stress by modulating NADPH metabolic flux represent a promising therapeutic avenue for MIRI.

The Toll like receptor (TLR) signaling pathway plays a critical role in innate immunity and serves as the first line of defense against pathogen-associated molecular patterns (PAMPs) and damage-associated molecular patterns (DAMPs). When PAMPs/DAMPs released from cells damaged by oxidative stress and ROS during ischemia/reperfusion (I/R) bind to TLRs, the receptors activate, form dimers, and recruit adapter proteins such as myeloid differentiation primary response 88 (MyD88). The activated MyD88 subsequently triggers downstream IRAK4/IRAK1/TRAF6/NF-κB signaling cascades, initiates pro-inflammatory gene transcription, and provokes excessive innate immune inflammatory responses. It has been reported that NOX is a downstream target of the TLR pathway and that NOX-derived ROS may be modulated by IRAK4/1 activity [13]. TLR activation enhances NOX-mediated ROS production and promotes inflammatory responses and host immune defense [14,15,16]. The TLR4/NOX2 signaling axis has been recognized as a key contributor to oxidative stress-induced inflammatory responses [17,18,19,20]. Importantly, emerging evidence suggests that TLR signaling not only regulates immune responses but also directly modulates cellular metabolic programs, including NADPH utilization and redox metabolism. Therefore, targeting the TLR signaling pathway may offer a redox-regulatory immune therapeutic strategy for MIRI, although the underlying molecular mechanisms remain incompletely understood.

MyD88 is a key adaptor molecule for almost all TLRs (except TLR3) [21]. Our previous studies have verified that the novel patented small-molecule MyD88 inhibitor TJ-M2010-5 effectively suppresses MyD88 homodimerization and blocks the TLR/MyD88/NF-κB signaling cascade, thereby exerting potent anti-inflammatory and immunomodulatory effects in multiple disease models [22,23,24,25,26,27,28,29,30]. Given the critical role of MyD88 in both immune activation and metabolic regulation, the present study was designed to investigate whether TJ-M2010-5 protects against MIRI by suppressing the MyD88–NF-κB–NOX/ROS cascade and preserving eNOS activity through restoration of NADPH redox homeostasis. This study reveals a novel redox-regulatory immune mechanism of MyD88-mediated MIRI pathogenesis and provides a promising targeted intervention strategy for clinical MIRI treatment.

## 2. Results and Discussion

### 2.1. Blocking the MyD88 Signaling Pathway with TJ-M2010-5 Reduces Myocardial Infarction in a Mouse MIRI Model

To determine whether pharmacological inhibition of the MyD88 signaling pathway confers cardioprotection against MIRI, we established a mouse I/R model by 30 min left anterior descending coronary artery (LCA) ligation followed by 24 h reperfusion. Male C57BL/6 mice were randomly divided into sham, I/R, Sham + TJ-M2010-5 (Sham-Inh) and TJ-M2010-5 pretreatment (Inhibitor-I/R) groups to examine whether TJ-M2010-5 alleviates myocardial infarction induced by MIRI. Baseline body weights were comparable across all groups (Sham: 22.3 ± 1.1 g; I/R: 22.8 ± 1.3 g; Sham-Inh: 22.5 ± 1.0 g; Inhibitor-I/R: 23.1 ± 0.9 g; *p* > 0.05 by one-way ANOVA). No perioperative mortality was observed; all mice recovered from anesthesia and survived until sacrifice at 24 h post-reperfusion.

**Figure 1:**
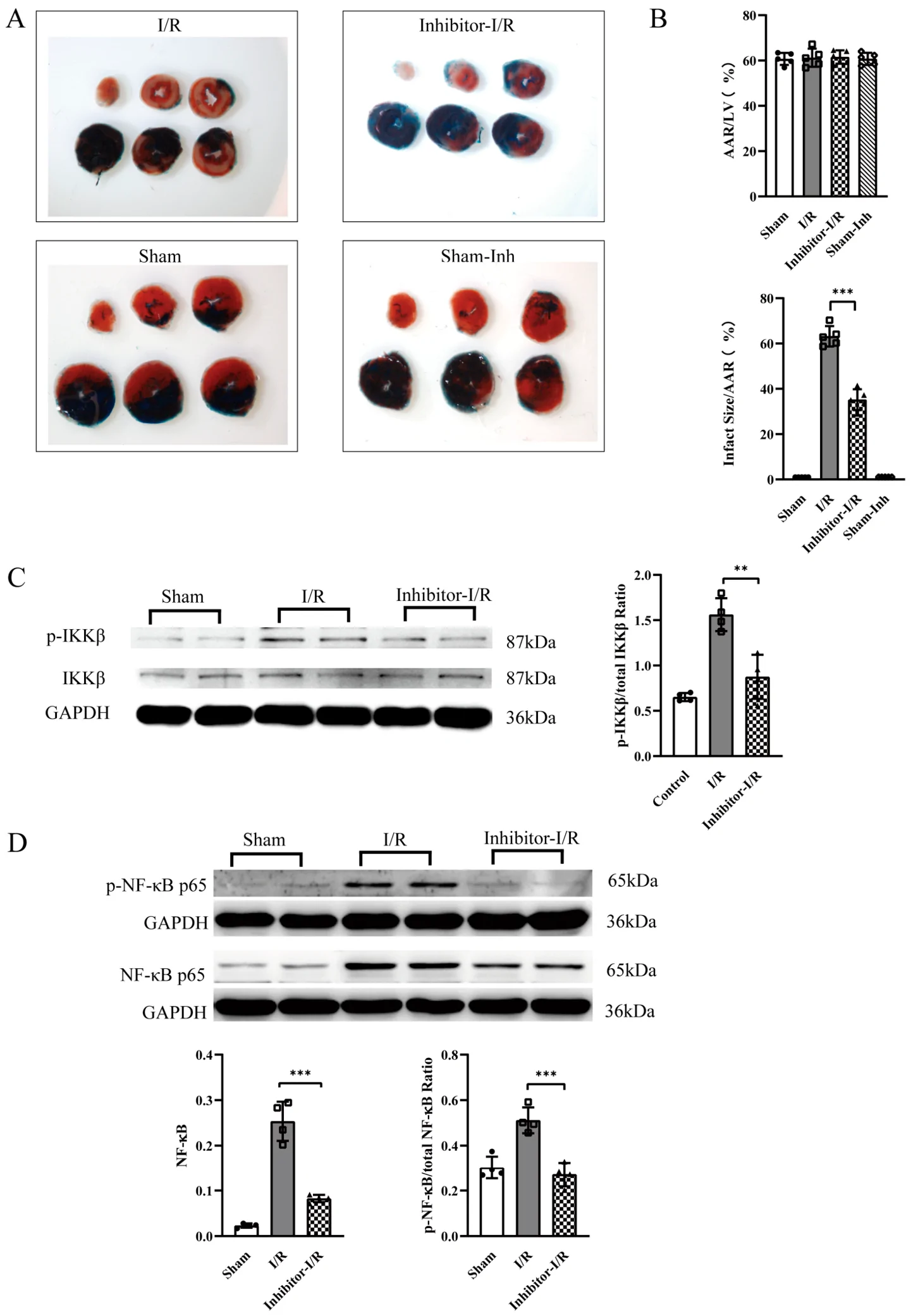
MyD88/NF-κB signaling pathway activation is involved in MIRI. (**A**,**B**) Assessment of myocardial infarction in mouse hearts subjected to 30 min LCA ligation followed by 24 h reperfusion, w/o administration of the MyD88 inhibitor TJ-M2010-5. Groups include Sham, Sham-Inh (Sham + TJ-M2010-5), I/R, and Inhibitor-I/R. (**A**) Representative heart sections stained with Evans blue and TTC after I/R. (**B**) AAR is expressed as a percentage of LV (AAR/LV). LV infarct size is expressed as a percentage of the AAR. Data are presented as means ± SD with individual data points overlaid. *N* = 5/group. Comparisons between I/R and Inhibitor-I/R were analyzed by unpaired two-tailed Student’s *t*-test; other comparisons by one-way ANOVA with Tukey’s post hoc test. *** *p* < 0.001. (**C**,**D**) Immunoblot analysis of total and phosphorylated forms of IKKβ (**C**) and NF-κB p65 (**D**) in cardiac homogenates from the AAR. Left panels (**C**) and top panels (**D**): Representative Western blots for IKKβ, p-IKKβ (Ser181), NF-κB p65, and p-NF-κB p65 (Ser536). Phospho-signals were normalized to matched total proteins from the same membrane; total proteins were normalized to GAPDH. Right panels (**C**) and bottom panels (**D**): Densitometric quantification of protein levels. Data are presented as means ± SD with individual data points overlaid. *N* = 4/group. ** *p* < 0.01, *** *p* < 0.001.

After reperfusion, hearts were stained with Evans Blue and 2,3,5-triphenyltetrazolium chloride (TTC) to quantify the area at risk (AAR) and infarct size. The AAR as a percentage of left ventricular (LV) area was similar between the I/R and Inhibitor-I/R groups (61.2 ± 4.0% vs. 61.6 ± 3.0%, respectively). However, TJ-M2010-5 treatment significantly reduced infarct size relative to AAR compared with the I/R group (35.2 ± 4.6% vs. 63.2 ± 4.5%; *p* < 0.0001, unpaired two-tailed Student’s *t*-test, *N* = 5/group), whereas the Sham-Inh group showed zero infarct size, indistinguishable from Sham (Figure 1A,B). Individual data points are overlaid on all bar graphs.

To further elucidate the underlying immunological mechanism by which TLR/MyD88 signaling mediates cardiac injury, we performed Western blot to detect the phosphorylation levels of IκB kinase β (IKKβ) and NF-κB p65 subunit in cardiac tissue, and evaluated NF-κB pathway activation. The ratios of p-IKKβ (Ser181)/total IKKβ, NF-κB p65/GAPDH, and p-NF-κB p65 (Ser536)/total NF-κB p65 were all significantly lower in the Inhibitor-I/R group than in the I/R group: 0.88 ± 0.24 vs. 1.56 ± 0.05 (*p* = 0.002), 0.082 ± 0.008 vs. 0.25 ± 0.04 (*p* = 0.0002), and 0.27 ± 0.05 vs. 0.51 ± 0.06 (*p* = 0.0008), respectively (*N* = 4/group) (Figure 1C,D).

These findings confirm that pharmacological inhibition of the MyD88/NF-κB innate immune signaling cascade confers robust cardioprotection against MIRI. Previous reports indicate that TLR signaling can transcriptionally up-regulate NOX1 via NF-κB [31], and that TLR-IRAK4-NF-κB signaling activation promotes p47phox phosphorylation and membrane translocation of gp91phox/NOX2 for complex assembly [32]. The present results align with these mechanisms, indicating that TLR-MyD88-NF-κB activation primarily enhances NOX subunit expression and activity during I/R. Notably, the ~44% relative reduction in infarct size achieved by MyD88 inhibition suggests that targeting the adaptor protein upstream of multiple NOX isoforms may offer broader efficacy than subtype-selective inhibition alone.

It is important to contextualize these pharmacological findings with genetic loss-of-function data. Published studies have established that MyD88 knockout mice are protected from ischemia–reperfusion injury across multiple organs [33,34]. In our preliminary study, MyD88-deficient mice indeed showed smaller infarct areas after MIRI. However, unlike acute pharmacological inhibition with TJ-M2010-5 in wild-type mice, germline knockout animals did not exhibit significant alterations in NOX2/NOX4 expression; instead, only inflammation-related genes were modulated. We attribute this discrepancy to well-documented compensatory developmental rewiring in germline knockout mice, which can obscure acute signaling events relevant to clinical disease. Consequently, acute pharmacological blockade with TJ-M2010-5—a peptidomimetic inhibitor specifically targeting MyD88 homodimerization—more faithfully recapitulates the pathophysiology of acute IRI and offers clearer translational relevance.

### 2.2. Blocking MyD88 Signaling Decreases ROS Generation in H5V After Hypoxia/Reoxygenation (H/R) In Vitro

To examine the impact of MyD88 signaling blockade on endothelial redox status and viability under ischemic stress, H5V mouse cardiac vascular endothelial cells (a transformed microvascular endothelial cell line) were subjected to H/R to mimic endothelial IRI.

H5V cells retain key phenotypic features of cardiac microvascular endothelium (CD31+, vWF+; Supplementary Figure S1A) and have been widely used in cardiac ischemia–reperfusion studies. PCR-based mycoplasma detection using primers targeting the conserved 16S rRNA region confirmed that the cells were free of mycoplasma contamination (Supplementary Figure S1B).

**Figure 2:**
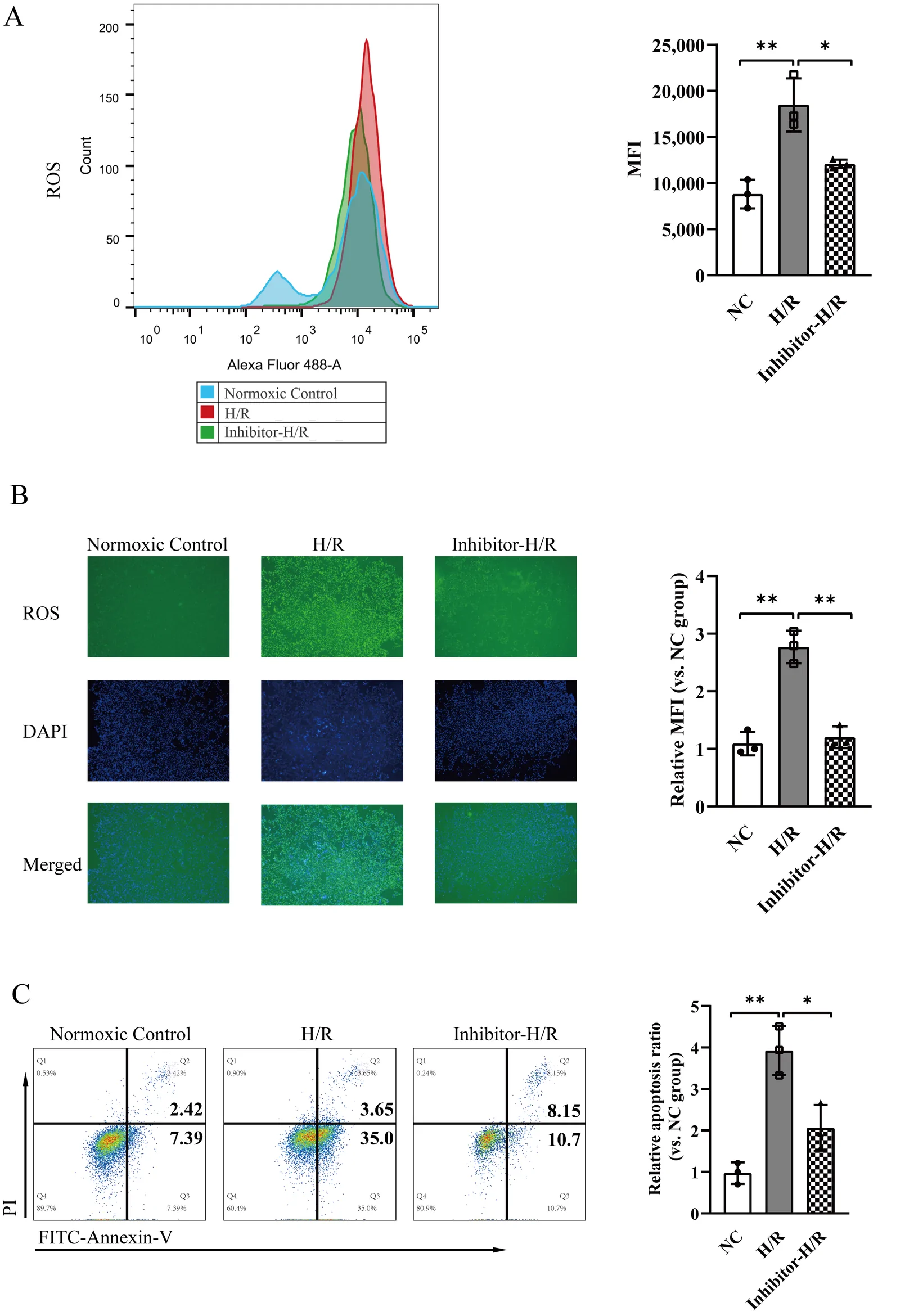
Effect of MyD88 signaling pathway activation on ROS production and apoptosis in H5V cells subjected to H/R. (**A**,**B**) ROS production under 6 h hypoxia/2 h reoxygenation, detected by DCFH-DA staining. (**A**) FCM analysis showing representative histograms (**left**) and quantification of MFI (**right**). (**B**) Representative fluorescence micrographs (**left**; green, ROS; blue, DAPI nuclei; Original magnification, 100×) and quantification of relative ROS MFI by ImageJ Software (v1.54u) with background subtraction (**right**). (**C**) Cell apoptosis under 24 h hypoxia/6 h reoxygenation, assessed by Annexin V-FITC/PI double staining and FCM. Representative dot plots (**left**) and quantification of total apoptotic ratio (Annexin V^+^/PI^−^ plus Annexin V^+^/PI^+^, corresponding to Q2 and Q3 quadrants; **right**). All quantitative data are from three independent biological replicates. Data are presented as means ± SD with individual data points overlaid. * *p* < 0.05, ** *p* < 0.01.

Cells were pretreated with TJ-M2010-5 before hypoxia exposure. Intracellular reactive oxygen species (ROS) levels were quantified by DCFH-DA staining coupled with flow cytometry (FCM), and cellular apoptosis was assessed using Annexin V-FITC/propidium iodide (PI) double-staining (*N* = 3 independent experiments). Consistent with the well-established pathogenic role of oxidative stress in IRI-induced endothelial dysfunction, H/R markedly promoted intracellular ROS accumulation in H5V cells. Flow cytometric analysis revealed that the mean fluorescence intensity (MFI) of ROS was significantly higher in the H/R group (18,482.67 ± 2885.06) than in the normoxic control group (NC, 8820.67 ± 1552.03; *p* = 0.007). This H/R-triggered elevation in ROS was significantly attenuated by TJ-M2010-5 pretreatment (12,089.33 ± 459.98; *p* = 0.02 versus the H/R group) (Figure 2A). These findings were further validated by fluorescence-microscopy-based measurements: the relative ROS MFI in the H/R group reached 2.77 ± 0.28-fold of the normoxic baseline value (1.09 ± 0.21; *p* = 0.001). In contrast, ROS production was markedly reduced to 1.20 ± 0.19-fold relative to the normoxic baseline in the inhibitor-pretreated group (*p* = 0.001 versus the H/R group) (Figure 2B). Flow cytometric analysis showed that the apoptotic ratio was significantly increased in H/R-treated H5V cells, which was 3.93 ± 0.59-fold higher than that of the NC (1.97 ± 0.26; *p* = 0.001). As expected, TJ-M2010-5 pretreatment prominently reversed H/R-induced apoptosis, decreasing the apoptotic rate to 2.06 ± 0.55-fold of the normal baseline (*p* = 0.02 vs. the H/R group) (Figure 2C; total apoptosis was defined as the sum of Annexin V^+^/PI^+^ and Annexin V^+^/PI^−^ populations, corresponding to Q2 and Q3 quadrants in flow cytometry dot plots). Taken together, inhibition of MyD88 signaling mitigates H/R-induced excessive ROS accumulation, maintains intracellular redox homeostasis, and thereby promotes H5V cell survival.

### 2.3. TJ-M2010-5 Inhibits NOX Expression and Activity

**Figure 3:**
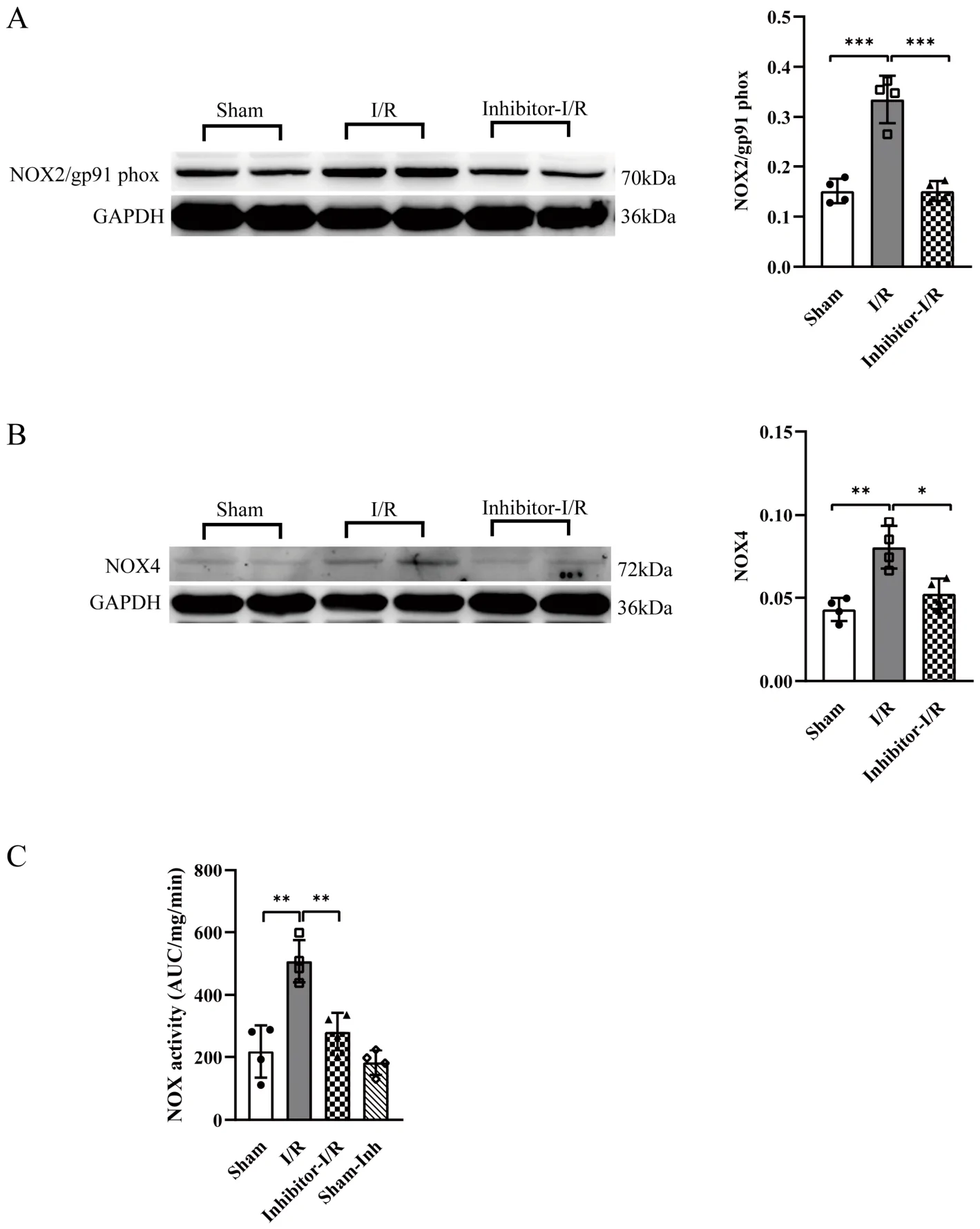
Effect of the MyD88 signaling pathway activation on NOX2 and NOX4 expression and activity during myocardial I/R. (**A**,**B**) Immunoblot analysis of NOX2/gp91 phox (**A**) and NOX4 (**B**) in cardiac homogenates from the AAR. Groups include Sham, I/R, and Inhibitor-I/R. **Left panels**: Representative Western blots. **Right panels**: Densitometric quantification. Data are presented as means ± SD with individual data points overlaid. *N* = 4/group. * *p* < 0.05, ** *p* < 0.01, *** *p* < 0.001. (**C**) NOX activity measured in cardiac homogenates from the AAR using a luminometric assay with lucigenin and NADPH. Groups include Sham, Sham-Inh, I/R, and Inhibitor-I/R. The AUC was normalized to sample protein content. Data are presented as means ± SD with individual data points overlaid. *N* = 4/group. ** *p* < 0.01.

Cardiac tissue within the AAR was harvested after in vivo I/R surgery. We used Western blot to detect NOX2 and NOX4 protein abundance (Figure 3A,B), and a lucigenin-dependent chemiluminescence assay to quantify total NOX activity (Figure 3C); the area under the curve (AUC) of luminescence signals was normalized to total protein content. NOX enzymes are the primary dedicated producers of ROS during IRI. Compared with sham non-ischemic myocardium, 30 min ischemia followed by 24 h reperfusion markedly upregulated cardiac NOX2 (2.2-fold) and NOX4 (1.9-fold) expression, as well as total NOX activity (2.3-fold) (*N* = 4/group). To determine whether enhanced NOX function following I/R is linked to TLR/MyD88 activation, mice were pretreated with TJ-M2010-5 (i.p.) 1 h before ischemia. TJ-M2010-5 administration significantly attenuated and restored toward sham levels the I/R-induced increase in myocardial NOX expression and activity (*p* = 0.0004, *p* = 0.01, and *p* = 0.007, respectively, vs. I/R group; *N* = 4/group). No significant changes in NOX activity were observed in the Sham-Inh group compared to Sham group.

These results provide in vivo and in vitro evidence that MyD88 acts downstream of TLR and upstream of NOX during I/R injury. By blocking MyD88 homodimerization, the inhibitor suppresses NOX2/4 expression, assembly, and enzymatic activity through an immunopharmacological mechanism, thereby attenuating NOX-dependent ROS overproduction.

### 2.4. TJ-M2010-5 Restores NADPH Levels in H5V Cells After H/R In Vitro

**Figure 4:**
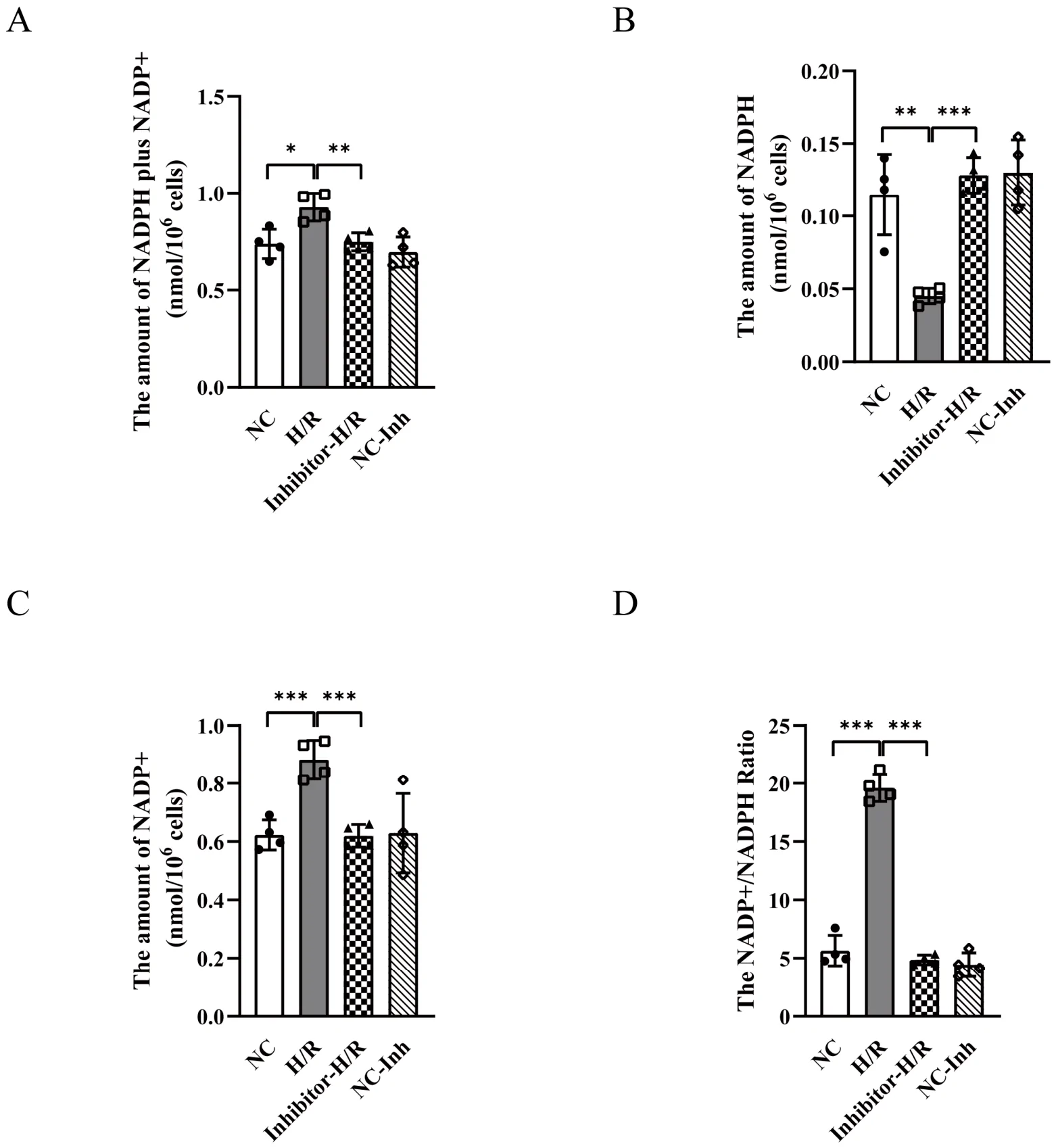
Intracellular levels of total NADP(H), NADPH, NADP^+^, and the NADP^+^/NADPH ratio in H5V cells after H/R w/o the MyD88 inhibitor TJ-M2010-5 treatment. H5V cells were subjected to 24 h hypoxia/6 h reoxygenation. Groups include untreated normoxic control (NC), H/R, H/R + TJ-M2010-5 (Inhibitor-H/R), and normoxic + TJ-M2010-5 (NC-Inh). (**A**) Total NADP(H), (**B**) NADPH, (**C**) NADP^+^, and (**D**) NADP^+^/NADPH ratio. Representative data are shown as means ± SD with individual data points overlaid. *N* = 4/group. * *p* < 0.05, ** *p* < 0.01, and *** *p* < 0.001.

To investigate whether MyD88 inhibition preserves NADPH redox homeostasis under H/R stress, intracellular NADPH and NADP^+^ levels in H5V were measured using a commercial NADP(H) quantification kit. Total NADP(H) and the NADP^+^/NADPH ratio were calculated to reflect cellular redox status. In untreated H5V, total NADP(H) (NADPH + NADP^+^) was 0.738 ± 0.076 nmol/mg protein (Figure 4A); NADPH was 0.115 ± 0.028 nmol/mg protein (Figure 4B); and NADP^+^ was 0.623 ± 0.051 nmol/mg protein (Figure 4C). The NADP^+^/NADPH ratio was 5.66 ± 1.32 (Figure 4D). H/R increased total NADP(H) to 0.927 ± 0.072 nmol/mg protein (*p* = 0.01 vs. untreated), decreased NADPH (*p* = 0.003 vs. untreated), and increased NADP^+^ (*p* = 0.0008 vs. untreated), leading to a markedly elevated NADP^+^/NADPH ratio (19.62 ± 1.18, *p* < 0.0001 vs. untreated). TJ-M2010-5 treatment during H/R restored the NADP^+^/NADPH ratio to 4.86 ± 0.41 (*p* < 0.0001 vs. H/R), reduced total NADP(H) to 0.748 ± 0.046 nmol/mg protein (*p* = 0.006 vs. H/R), recovered NADPH (*p* < 0.0001 vs. H/R), and lowered NADP^+^ (*p* = 0.0005 vs. H/R). The Inhibitor-H/R NADP^+^/NADPH ratio (4.86 ± 0.41) was statistically indistinguishable from the untreated control (5.66 ± 1.32; *p* > 0.05 by Tukey’s post hoc test) and well within inter-assay measurement variation, indicating restoration to physiological baseline rather than oversuppression. Normoxic cells treated with TJ-M2010-5 alone showed no significant alterations in NADP(H) pools or redox ratio compared to untreated controls.

From a redox homeostasis perspective, our findings reveal that MyD88 signaling blockade not only suppresses inflammatory responses but also restores cellular NADPH redox balance. This dramatic shift in the NADP+/NADPH redox couple indicates that blocking electron transfer from NADPH to NOX for ROS generation preserves NADPH availability for physiological antioxidant pathways and eNOS-dependent NO production. While these steady-state pool measurements demonstrate restored redox homeostasis, true metabolic reprogramming—defined as altered pathway flux—can only be confirmed through future [U-13C]-glucose tracing and LC-MS/MS flux analysis.

### 2.5. Blocking MyD88 Signaling Pathway Enhances eNOS Activity and Preserves NO Bioavailability

**Figure 5:**
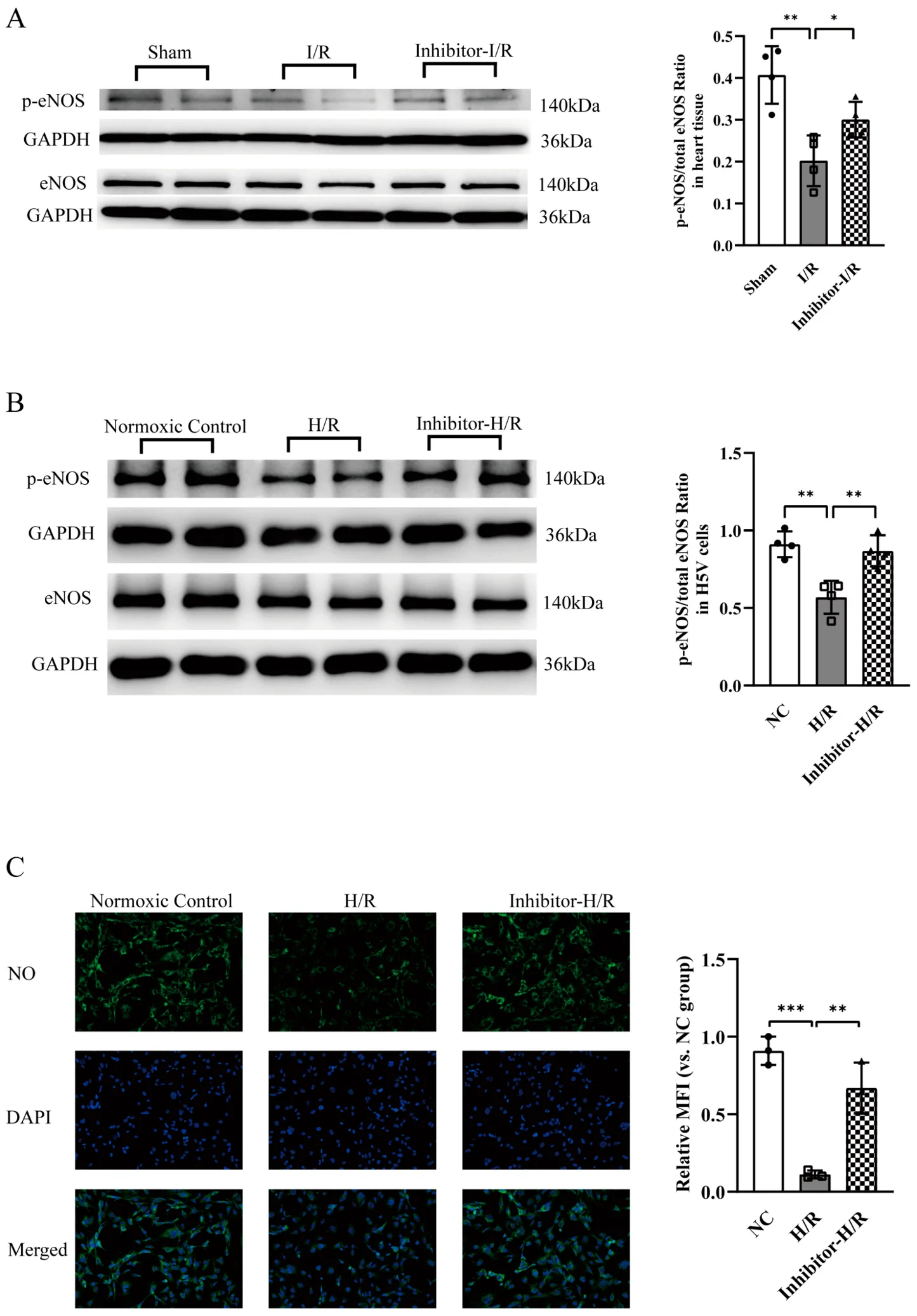
Inhibition of the MyD88 signaling pathway promotes eNOS phosphorylation and preserves NO bioavailability during myocardial I/R and H5V cells’ H/R. (**A**,**B**) Immunoblot analysis of total eNOS and its phosphorylated form (phosphor-eNOS Ser1177) in cardiac homogenates from the AAR (**A**) and in H5V cell lysates (**B**). H5V cells were subjected to 24 h hypoxia/6 h reoxygenation. Left panels: Representative Western blots for eNOS and p-eNOS. Phospho-signals were normalized to matched total proteins from the same membrane; total proteins were normalized to GAPDH. Right panels: Densitometric quantification. Data are presented as means ± SD with individual data points overlaid. *N* = 4/group. (**C**) Quantification of intracellular NO bioavailability by DAF-FM-DA fluorescent staining in H5V cells subjected to 6 h hypoxia/2 h reoxygenation. **Left**: Representative fluorescence images (green, NO; blue, nuclei; original magnification, 200×). **Right**: Quantification of relative NO MFI by ImageJ Software (v1.54u) with background subtraction. Experiments were independently repeated three times. Data are presented as means ± SD with individual data points overlaid. * *p* < 0.05, ** *p* < 0.01, and *** *p* < 0.001.

To assess whether MyD88 inhibition preserves eNOS functional activity, Western blot was conducted on AAR cardiac tissue and H/R-treated H5V lysates to detect total eNOS and its activating phosphorylation at Ser1177, an indicator of nitric oxide (NO)-dependent eNOS function (Figure 5A,B). Endothelium-derived NO generated by eNOS acts as a critical vasoprotective molecule. Functionally, eNOS shuttles electrons from NADPH via FAD/FMN within its reductase domain to the heme moiety of the oxygenase domain. Given that NADPH serves as an indispensable electron donor for eNOS, we compared the p-eNOS (Ser1177)/total eNOS ratio across all groups. Total eNOS levels were similar among groups after I/R or H/R, w/o TJ-M2010-5 treatment. However, the p-eNOS (Ser1177)/total eNOS ratio decreased significantly after I/R or H/R compared with baseline (~50% and ~38% reduction, respectively). In contrast, TJ-M2010-5 treatment largely prevented these decreases, limiting the reductions to only ~26% and ~5% after reperfusion/reoxygenation, whereas the Sham-Inh group showed no deviation from Sham controls.

Since p-eNOS (Ser1177) only serves as an indirect surrogate marker for eNOS catalytic activity, we further performed DAF-FM-DA fluorescent staining to directly quantify intracellular NO bioavailability in H/R-challenged H5V cells (Figure 5C). Quantitative analysis of MFI demonstrated that intracellular NO levels were markedly reduced in H/R-injured H5V cells relative to NC cells, with the H/R group exhibiting only 12.3% of the NO level observed in the NC group. Importantly, TJ-M2010-5 pretreatment significantly restored intracellular NO content in H/R-exposed H5V cells to levels comparable to the NC group (*p* > 0.05 vs. NC group), confirming that MyD88 inhibition preserves functional NO production.

I/R leads to eNOS uncoupling, a key event in the sustained ROS amplification and NO deficiency. We noted a 50% decrease in p-eNOS-Ser1177 after myocardial I/R, whereas TJ-M2010-5 limited this decrease to 26%, an effect coincident with restored NADPH abundance and rescued intracellular NO levels. This leads us to propose the hypothesis of “NADPH-eNOS coupled redox restoration”: inhibition of the TLR/MyD88-NOX axis → increased NADPH availability → enhanced electron supply to the eNOS reductase domain → maintenance of heme-Fe^2+^ in a reduced state → reduced superoxide anion leakage → restored NO generation. This concept is supported by reports that supplementation of NADPH can reverse eNOS uncoupling induced by H_2_O_2_ [35,36,37,38]. This redox coupling hypothesis positions NADPH not merely as a biochemical cofactor but as a central metabolic hub integrating innate immune signaling, cellular redox balance, and vascular endothelial function.

### 2.6. Direct Evidence for On-Target MyD88 Engagement by TJ-M2010-5 in MIRI

To provide direct evidence that TJ-M2010-5 engages MyD88 within the diseased tissue rather than acting through unrelated mechanisms, we performed co-immunoprecipitation (Co-IP) assays using AAR myocardial tissue subjected to ischemia/reperfusion, with in vivo DSP cross-linking to stabilize transient MyD88-MyD88 interactions. We found that myocardial I/R markedly increased MyD88 homodimerization (3.2-fold vs. Sham, *p* < 0.001), and this enhancement was significantly attenuated by TJ-M2010-5 pretreatment (1.4-fold vs. Sham; *p* < 0.01 vs. I/R), while total MyD88 expression remained unchanged (Supplementary Figure S2A). These data demonstrate that TJ-M2010-5 reaches its intended target in the ischemic heart and functionally disrupts MyD88 homodimerization.

The selectivity of TJ-M2010-5 for MyD88 over other homodimerization-dependent adaptors is supported by our previously published structural data (Xie et al., JNCI 2016, [22]). Molecular docking revealed that TJ-M2010-5 specifically binds to the box3 region within the MyD88 TIR domain, particularly the I179 residue on the BB loop, and interacts with surrounding loop regions (alphaE, betaD, betaC, DD loop, and EE loop). This binding pocket is structurally distinct from the corresponding regions in other TIR-domain-containing adaptors such as TRIF, TRAM, and TIRAP/MAL, making off-target binding to these molecules highly unlikely. In addition, co-immunoprecipitation experiments in HEK293 cells demonstrated that TJ-M2010-5 inhibits MyD88 homodimerization in a concentration-dependent manner without affecting the dimerization of other tested adaptor proteins.

To definitively rule out direct off-target effects on NOX2 or eNOS, we performed cell-free enzymatic assays using purified recombinant proteins. TJ-M2010-5 (up to 50 μM) did not inhibit recombinant NOX2 activity (measured by WST-1 reduction at 440 nm) (Supplementary Figure S2B) nor did it activate or inhibit recombinant eNOS activity (measured by DAF-2 DA fluorescence at Ex/Em 490/520 nm) (Supplementary Figure S2C). The positive controls DPI (for NOX2) and L-NAME (for eNOS) exhibited expected inhibition, confirming assay validity. In summary, the combined structural, biochemical, and in vivo functional evidence strongly supports the high specificity of TJ-M2010-5 for MyD88. Its effects on the NOX/ROS-eNOS axis in MIRI are mediated through an on-target, MyD88-dependent indirect mechanism rather than through direct off-target binding to NOX2 or eNOS.

Recent evidence indicates that myeloid fatty-acid synthesis via ACLY and FASN drives post-myocardial infarction fibrosis through epigenetic mechanisms linked to macrophage metabolic reprogramming, providing direct evidence that immune-associated metabolic regulators can modulate metabolic flux and influence cardiac outcomes following ischemic injury [39]. Additionally, broader perspectives on NOS isoform biology and NO-mediated regulation of neutrophil ontogeny and function complement the present focus on endothelial NOS, highlighting the versatile roles of nitric oxide signaling across innate immune cell lineages [40].

### 2.7. Limitations

We acknowledge limitations of the current study: future MyD88 overexpression rescue experiments are required to genetically validate this signaling cascade. Serial echocardiography, which is important for evaluating cardiac diastolic function, was not performed in the present work due to the unavailability of specialized equipment; these functional assessments will be implemented in follow-up studies when experimental conditions permit. Moreover, formal a priori sample-size power analysis was not performed. However, animal group sizes (*N* = 4–5) were selected based on our prior laboratory experience and pilot data. The large biological effect size observed in this study provides acceptable statistical power with this sample size. Genetic STR-based authentication of the H5V transformed endothelial cell line has also not been completed. These constitute additional methodological limitations of this work.

## 3. Materials and Methods

### 3.1. In Vivo Myocardial I/R and Measurement of Myocardial Infarction

In vivo myocardial I/R and infarct size measurements were performed in male C57B/6 mice (8-week-old, BEIJING HFK BIOSCIENCE Co., LTD, Beijing, China) as described previously with slight modification [41,42]. Mice were randomly allocated to treatment groups by a separate investigator prior to surgery. Infarct size measurement by computerized planimetry was performed by investigators blinded to group allocation. Mice were anesthetized by intraperitoneal injection of pentobarbital sodium (60 mg/kg). A midline neck incision was made, and the trachea was exposed and intubated with a cannula connected to a rodent ventilator (Kent Scientific, Inc., Torrington, CT, USA). Mice were ventilated with room air at 100–120 breaths/min and a tidal volume of 0.15 mL. Body temperature was maintained at 37.5 °C using a heating pad. A left thoracotomy was performed in the third intercostal space, followed by pericardiotomy. LCA was encircled with a 9–0 silk suture to form a slipknot 2.0 mm below the tip of the left atrial appendage; a small segment of PE-10 tubing was placed under the suture to facilitate release upon reperfusion. LCA occlusion was confirmed by visible pallor and akinesia of the anterior wall. Reperfusion was initiated by releasing the slipknot and removing the tubing, and was confirmed by epicardial hyperemia. The suture was left for later re-ligation and staining, and the chest was closed in layers.

After 24 h of reperfusion, mice were euthanized; hearts were rapidly excised and flushed with heparinized PBS via aorta, and then incubated with 1.5%TTC (Sigma-Aldrich, St. Louis, MO, USA) for 5 min at 37 °C to demarcate viable and nonviable myocardium within the AAR. The LCA was re-occluded, and 2% Evans Blue was injected via the aorta to delineate the non-ischemic region. Hearts were frozen, serially sectioned (2 mm thick), and fixed in 10% formalin. Both sides of each slice were photographed, and the AAR and infarct area were measured by computerized planimetry using image analysis software (MetaVue, version 6.0).

### 3.2. Animal Groups and Treatment Protocols

Male C57B/6 mice were randomly allocated into four groups (*N* = 4-5/group):

• Sham control: subjected to 0 min ischemia.
• I/R: subjected to 30 min ischemia followed by 24 h reperfusion.
• Inhibitor-I/R: treated with TJ-M2010-5 (50 mg/Kg, i.p.) 1 h before ischemia.
• Sham-Inh: treated with TJ-M2010-5 (50 mg/kg, i.p.) 1 h before sham surgery.

The in vivo dose of 50 mg/kg was determined from preliminary dose–response experiments in mice, where 50 mg/kg was identified as the minimum effective dose that provided protection against MIRI without eliciting toxicity (liver and kidney functions remained normal). TJ-M2010-5 was dissolved in sterile water for injection.

### 3.3. H5V Cell Culture and H/R In Vitro

H5V mouse cardiac endothelial cells (a transformed microvascular endothelial cell line) were purchased from the China Center for Type Culture Collection (CCTCC, Wuhan, China).

Cells were cultured in DMEM (GIBCO, Thermo Fisher Scientific, Shanghai, China) supplemented with 5% fetal bovine serum (Yeasen Biotec, Shanghai, China) at 37 °C in a humidified incubator with 95% air and 5% CO_2_.

To simulate cardiac endothelial cells IRI in vitro, H/R was performed: for ROS and NO assays, H5V cells were exposed to hypoxia for 6 h in a hypoxic incubator (1% O_2_, 5% CO_2_, balance N_2_) at 37 °C, and then reoxygenated for 2 h under normoxic conditions (95% air, 5% CO_2_); for apoptosis assays, NADP(H) assays and Western blot, H5V cells were exposed to hypoxia for 24 h, and then reoxygenated for 6 h under normoxic conditions. In the Inhibitor-H/R group, H5V cells were pretreated with TJ-M2010-5 (20 μM, dissolved in sterile water) 30 min before hypoxia.

This in vitro concentration was selected based on our previous in vitro pharmacokinetic studies [22,27], which demonstrated that 20 μM is the minimum effective concentration for inhibiting MyD88 dimerization without causing cytotoxicity.

### 3.4. Western Blot Analysis

Heart tissues from the AAR were frozen and homogenized, and cells were lysed in ice-cold RIPA buffer containing phosphatase inhibitors. Equal amounts of protein from tissue or cell lysates were separated by SDS-PAGE using 10% gels for IKKbeta, NF-kappaB p65, NOX4, NOX2/gp91phox, and GAPDH, and 8% gels for eNOS and transferred to PVDF membranes. Membranes were blocked with 5% skim milk in TBST for 1 h at room temperature and subsequently incubated with primary antibodies overnight at 4 °C. Detailed antibody information, including catalogue numbers, dilutions, and RRIDs where available, is provided in Supplementary Table S1. After washing, membranes were incubated with HRP-conjugated goat anti-rabbit IgG (Jackson ImmunoResearch Lab) for 1 h at room temperature (RT). Signals were detected with Chemiluminescence Plus reagent (Powerful Bio), visualized using a BioSpectrum 815 imaging system, and quantified with Image-Pro Plus software (v10). GAPDH served as a loading control.

Phospho-protein signals were normalized to their matched total protein signals from the same membrane (after stripping and re-probing), and total protein signals were normalized to GAPDH. Western blot densitometric analysis was performed by investigators blinded to group allocation.

### 3.5. ROS and NO Assays

After 6 h hypoxia and 2 h reoxygenation, H5V cells were incubated with either the ROS probe DCFH-DA (Servicebio, Wuhan, China) or the NO probe DAF-FM-DA (Servicebio, China) at 37 °C for 30 min. Cells were then fixed with 4% paraformaldehyde for 20 min, washed with PBS, and stained with Hoechst 33342 (Beyotime, Beijing, China) for nuclear counterstaining. Fluorescence images were acquired under a microscope, and fluorescence intensity was quantified as MFI using ImageJ Software (v1.54u) with background subtraction. Additionally, ROS levels were analyzed by FCM using FlowJo software (V10.0).

### 3.6. Apoptosis Detection

After 24 h hypoxia and 6 h reoxygenation, H5V cell apoptosis was assessed using an Annexin V-FITC/PI apoptosis kit (Multisciences Biotech Co., Ltd., Hangzhou, China). Briefly, cells were harvested by trypsinization, washed with cold PBS, centrifuged at 300× *g* for 5 min, and resuspended in Binding Buffer. Then, 500 μL of cell suspension was incubated with 5 μL of FITC-Annexin V and 10 μL of PI for 5 min at RT in the dark. Samples were analyzed by FCM using FlowJo. Apoptosis was defined as total Annexin V+ cells (early and late apoptotic populations, i.e., the sum of Q2 and Q3 quadrants).

### 3.7. NOX Activity Measurement

NOX activity was measured in heart AAR tissue. Tissue was cryo-grinded in liquid nitrogen, homogenized in ice-cold PBS, and centrifuged at 3000× *g* for 20 min at 4 °C. The supernatant was collected and placed in a luminometer, and lucigenin (5 μM) was added. After thermal equilibration (15 min, 37 °C), the reaction was initiated by injecting NADPH (100 μM). Kinetics were recorded, and the AUC was calculated using GraphPad Prism 8. Results were normalized to protein content.

### 3.8. Determination of NADPH and NADP^+^ Levels

NADPH and NADP^+^ levels were measured using a CoenzymeII NADP(H) Content Assay Kit (Solarbio Life Sciences, Beijing, China) according to the manufacturer’s instructions. Briefly, 5 × 10^6^ H5V cells were dissolved in 500 μL of 0.01 M NaOH (for NADPH) or 0.01 M HCl (for NADP^+^). Extracts were sonicated for 1 min, boiled for 5 min, cooled on ice, and centrifuged at 10,000× *g* for 10 min at 4 °C. Then, 200 μL of supernatant was transferred to a new tube, mixed with an equal volume of alkaline (for NADPH) or acidic (for NADP^+^) extraction solution to neutralize pH, and centrifuged again. The supernatant was kept on ice for assay. Standards (1.25 nmol/mL NADPH or NADP^+^) were prepared in distilled water. Samples, reagents and standards were added to a 96-well plate in the prescribed proportion. After incubation at RT for 20 min, absorbance was read at 570 nm with a microplate reader. Levels of NADPH or NADP^+^ (nmol/10^6^ cells) were calculated as: 1.25 × (sample absorbance − blank absorbance) ÷ (standard absorbance − blank absorbance).

### 3.9. Statistical Analysis

Data are presented as mean ± standard deviation (SD). All bar graphs include overlaid scatter plots showing individual data points. Statistical comparisons between groups were performed using one-way ANOVA, followed by Tukey’s post hoc test for multiple-group comparisons, and unpaired two-tailed Student’s *t*-test for strictly two-group comparisons. A *p*-value < 0.05 was considered statistically significant.

All statistical analyses were performed using GraphPad Prism 8.0 software.

## 4. Conclusions

The concept of immune metabolism has emerged as a paradigm-shifting framework in cardiovascular research. Our work demonstrates that MyD88, traditionally viewed as an immune adaptor, functions as a critical redox-regulatory node that controls NADPH flux and redox homeostasis. By targeting MyD88 homodimerization, TJ-M2010-5 represents a novel class of immune–redox modulators that bridge innate immunity and cellular metabolism. This approach differs fundamentally from conventional anti-inflammatory strategies by directly restoring redox homeostasis rather than merely suppressing immune responses.

From an immunopharmacological perspective, TJ-M2010-5 represents a novel class of innate immune modulators that target adaptor protein homodimerization rather than receptor-ligand binding. Unlike TLR4 antagonists (e.g., eritoran) that block specific PAMP/DAMP recognition, or NF-κB inhibitors that may cause broad immunosuppression, MyD88 homodimerization inhibitors offer a more nuanced approach by disrupting signal transduction at a critical node shared by multiple TLRs (except TLR3). Moreover, by simultaneously modulating immune signaling and redox pathways, TJ-M2010-5 exemplifies the emerging paradigm of redox pathway modulation in immunopharmacology. This positions TJ-M2010-5 as a potential broad-spectrum anti-inflammatory agent with applications extending beyond MIRI to other TLR-driven inflammatory and ischemic conditions characterized by redox dysregulation.

In conclusion, pharmacological inhibition of MyD88 by the novel small-molecule inhibitor TJ-M2010-5 attenuates MIRI. This cardioprotection is achieved through a multi-pronged immunopharmacological mechanism: (1) suppression of IKKβ/NF-κB innate immune signaling, (2) inhibition of NOX2/NOX4-derived ROS production, (3) restoration of NADPH redox homeostasis and redox balance, and (4) preservation of eNOS Ser1177 phosphorylation and endothelial function. The TLR/MyD88-NOX/ROS-eNOS axis represents a promising integrated molecular redox-regulatory therapeutic target for MIRI and potentially other TLR-driven inflammatory cardiovascular diseases.

## Data Availability

The authors confirm that the data supporting the findings of this study are available within the article and its Supplementary Materials, and from the corresponding author upon reasonable request.

## Supplementary Materials

The following supporting information can be downloaded at: https://www.mdpi.com/article/10.3390/ijms27188299/s1.
