# Supplementary material for: Blocking MyD88 Signaling Pathway Protects Against Myocardial Ischemia Reperfusion by Inhibiting NOX/ROS Pathway and Enhancing eNOS Activity
Source: Int J Mol Sci. 2026 Sep 17;27(18):8299. doi: 10.3390/ijms27188299 (PMC13607957; doi:10.3390/ijms27188299)
Supplement: Supplementary file 1 [file ijms-27-08299-s001.zip › supplementary.pdf]

Supplementary Figure S1

A

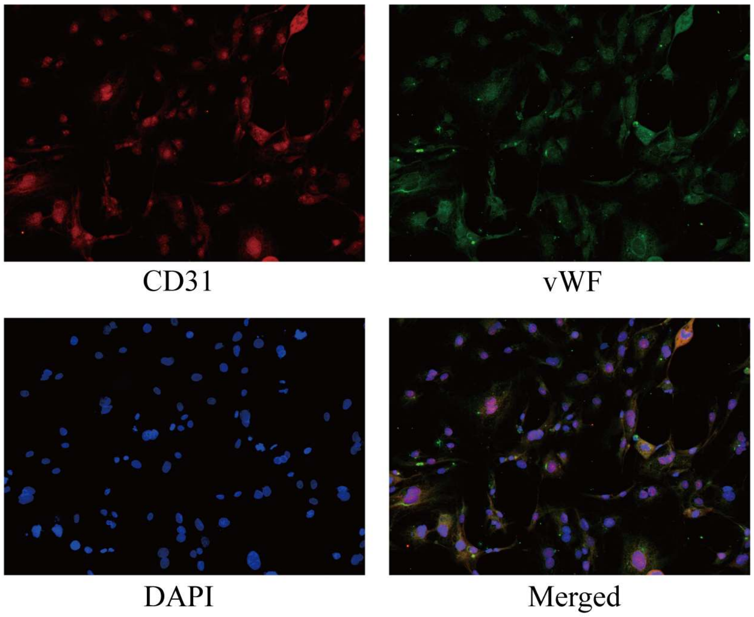

B

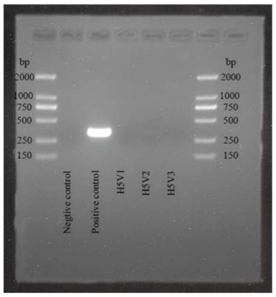

Scheme S1. Characterization and quality control of H5V cells. (A) Immunofluorescence double-staining confirming endothelial identity ($CD31^{+}$, red; vWF^{+}, green) of H5V mouse cardiac endothelial cells. Nuclei were counterstained with DAPI (blue). Original magnification, 200×. (B) PCR-based mycoplasma detection using primers targeting the conserved 16S rRNA region of common mycoplasma species, confirming absence of mycoplasma contamination in the cell batch used. Negative (Neg) and positive (Pos) controls were included.

Supplementary Figure S2

A

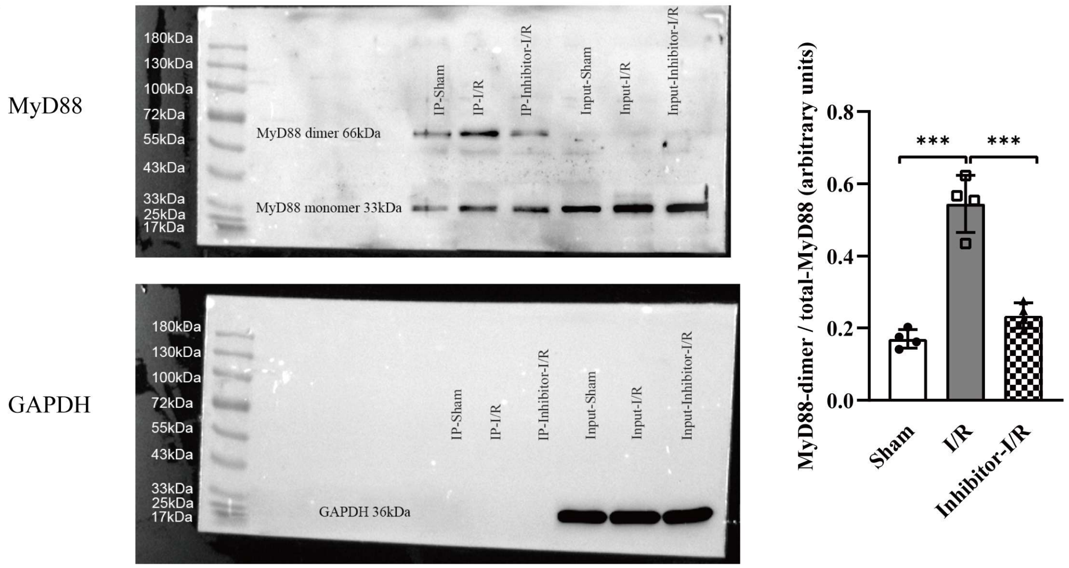

B

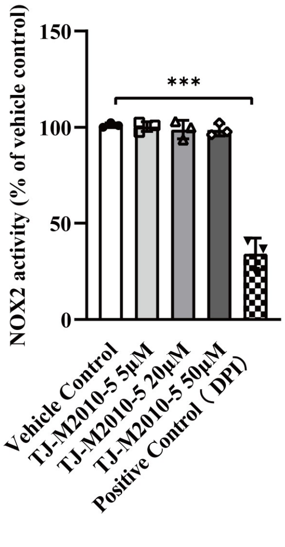

C

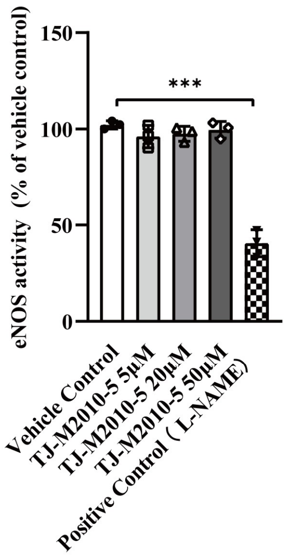

Figure S2. Direct evidence for on-target MyD88 engagement and off-target exclusion of TJ-M2010-5. (A) Co-immunoprecipitation (Co-IP) assay for MyD88 homodimerization in AAR myocardial tissue after in vivo DSP cross-linking. Representative immunoblots (left) show MyD88 dimer (~66 kDa) and input controls; densitometric quantification (right) of MyD88 dimer normalized to total MyD88 in input. N = 4/group. ***$P < 0.001$. (B) Cell-free recombinant NOX2 activity assay. TJ-M2010-5 (5–50 μM) did not inhibit recombinant NOX2 activity (WST-1 reduction at 440 nm); diphenyleneiodonium chloride (DPI, 10 μM) served as positive control. (C) Cell-free recombinant eNOS activity assay. TJ-M2010-5 (5–50 μM) did not activate or inhibit recombinant eNOS activity (DAF-2 DA fluorescence at Ex/Em 490/520 nm); Nω-nitro-L-arginine methyl

ester (L-NAME, 1 mM) served as positive control. All measurements were performed in triplicate. Data are presented as means ± SD. ***P < 0.001 versus vehicle control.

Table S1. Detailed antibody information including catalogue numbers, dilutions, and RRIDs.

Antibody	*Vendor*	*catalogue numbers*	*dilutions*	*RRIDs*
IKKβ	Affinity Biosci.	AF6014	1:500	AB_2834948
p-IKKβ (Ser181)	Affinity Biosci.	AF3013	1:500	AB_2834452
NF-kB p65	Huabio.	ET1603-12	1:5000	—
p-NF-kB p65 (Ser536)	CST	3033T	1:1000	—
NOX2/gp91 phox	Abclonal	A1636	1:500	—
NOX4	Abclonal	A2201	1:250	—
eNOS	Santa Cruz Biotec.	SC-654	1:200	AB_631423
p-eNOS (Ser1177)	Abclonal	AP0414	1:500	—
GAPDH	Huabio.	ET1601-4	1:5000	—
